# Supplementary material for: Phylogenomic Characterization of Lopma Virus and Praja Virus, Two Novel Rodent-Borne Arteriviruses
Source: Viruses. 2021 Sep 15;13(9):1842. doi: 10.3390/v13091842 (PMC8473226; doi:10.3390/v13091842)
Supplement: Supplementary file 1 [file viruses-13-01842-s001.zip › Supplementary_Figure_S1.pdf]

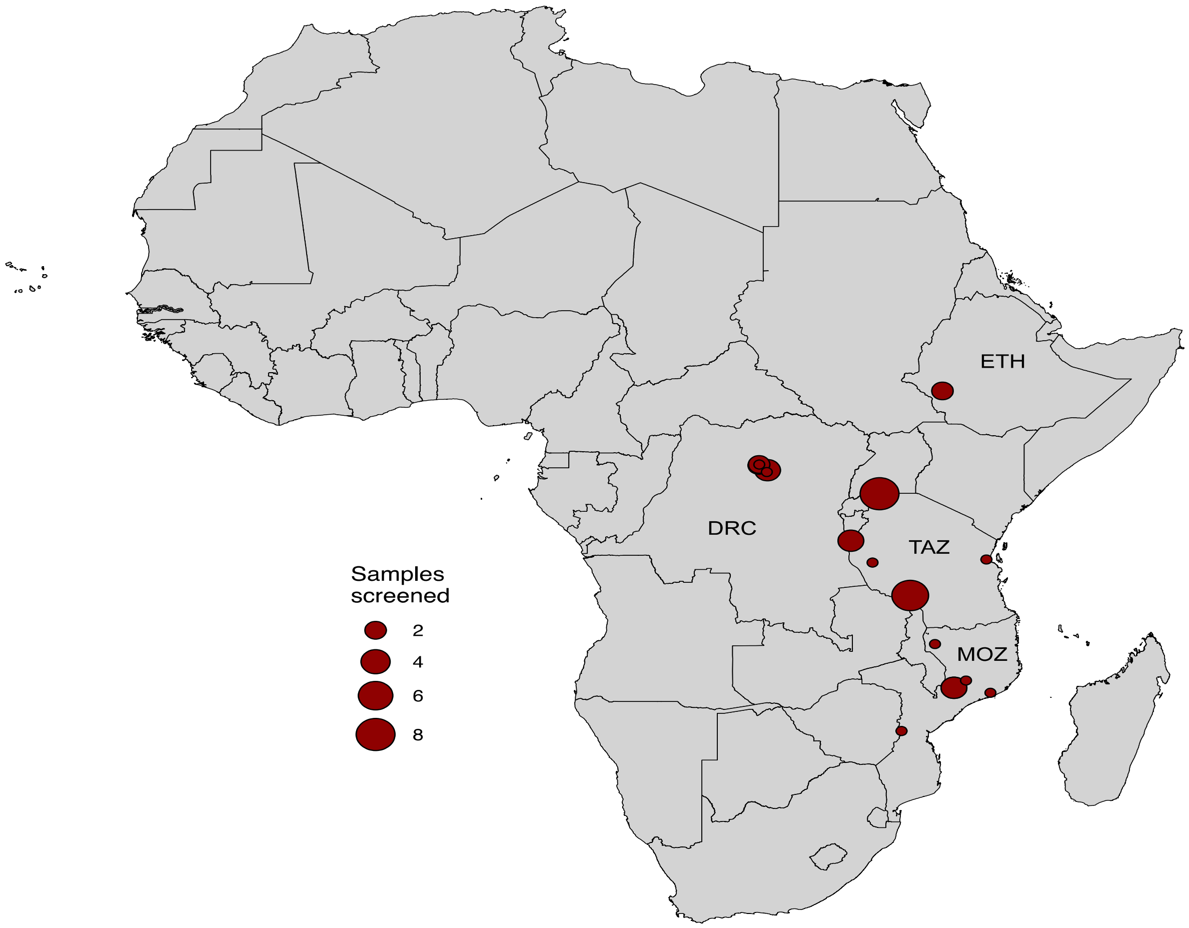

Supplementary Figure S1: Spatial distribution of our screened samples. Map of Africa indicating sampling sites of our specimen collection within the Democratic Republic of the Congo (DRC), Ethiopia (ETH), Mozambique (MOZ) and Tanzania (TAZ). Red circles denote the number of rodent individuals screened in each locality. Circle sizes are proportional to the number of samples, ranging from 1 to 8 rodent specimens per site.
