# Supplementary material for: Phylogenomic Characterization of Lopma Virus and Praja Virus, Two Novel Rodent-Borne Arteriviruses
Source: Viruses. 2021 Sep 15;13(9):1842. doi: 10.3390/v13091842 (PMC8473226; doi:10.3390/v13091842)
Supplement: Supplementary file 1 [file viruses-13-01842-s001.zip › Supplementary_Figure_S2.pdf]

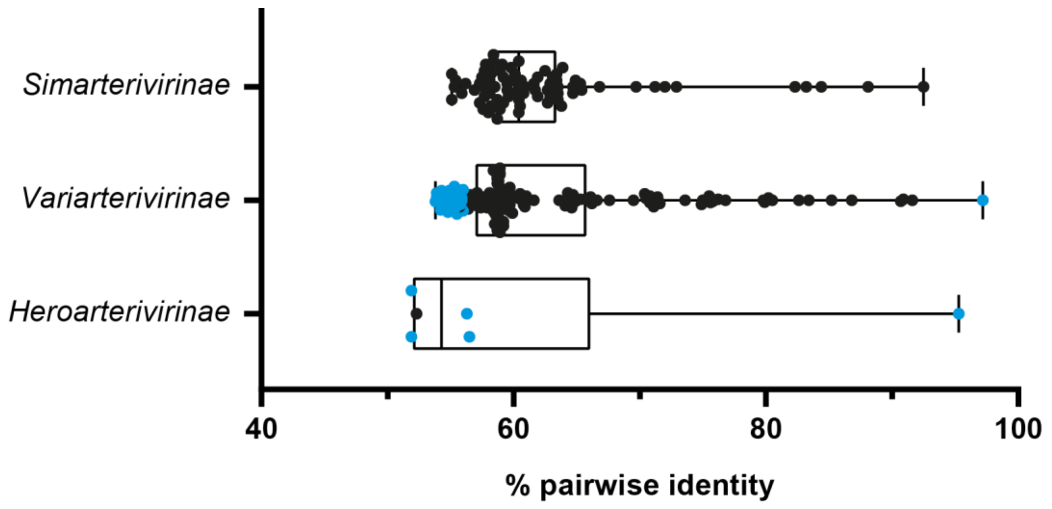

Supplementary Figure S2: Comparison of the intra-subfamily pairwise distance between members of the multimember subfamilies within the family *Arteriviridae*. Figure based on the values shown in Supplementary Table S4. Each dot represents the pairwise identity between two sequences, based on an alignment covering highly conserved parts of the ORF1ab polyprotein (nsp4 + nsp8-10). Values obtained from comparisons using Lopma virus or Praja virus are colored in blue.
